# Does preoperative neutrophil to lymphocyte or platelet to lymphocyte ratios have a role in predicting borderline ovarian tumors?

**DOI:** 10.1186/s13048-016-0283-2

**Published:** 2016-11-08

**Authors:** Ghanim Khatib, Cenk Soysal, Cihan Çetin, Ümran Küçükgöz Güleç, Ahmet Barış Güzel, Nadi Keskin, Mehmet Ali Vardar, Derya Gümürdülü

**Affiliations:** 1 Department of Obstetrics and Gynecology, Faculty of Medicine, Çukurova University, Adana, Turkey; 2 Department of Obstetrics and Gynecology, Faculty of Medicine, Dumlupınar University, Kütahya, Turkey; 3 Department of Pathology, Division of Gynecologic Pathology, Faculty of Medicine, Çukurova University, Adana, Turkey; 4 Department of Obstetrics and Gynecology, Division of Gynecologic Oncology, Faculty of Medicine, Çukurova University, Adana, 01330 Turkey

**Keywords:** Borderline ovarian tumors, Neutrophil to lymphocyte ratio, Platelet to lymphocyte ratio

## Abstract

**Background:**

to investigate the value of using preoperative neutrophil to lymphocyte and platelet to lymphocyte levels in the patients of borderline ovarian tumors.

**Methods:**

During the period between January 2002 and December 2015, the pathology reports and archival files of the Gynecologic Oncology Department of Çukurova University Medical Hospital and the Gynecologic Oncology Department of Dumlupınar University, Evliya Çelebi Education and Research Hospital were retrospectively reviewed, and 144 patients of borderline ovarian tumor (as the study group) and 123 patients of serous cystadenoma (as the control group) were determined for eligibility in this study. Data regarding age, menopausal status, preoperative ultrasound findings, ca125 and complete blood counts were reviewed. Neutrophil to lymphocyte and platelet to lymphocyte ratios were calculated and these parameters were statistically compared between the groups.

**Results:**

There was a statistically significant difference between the groups according to neutrophil count, platelet count, neutrophil to lymphocyte ratio and platelet to lymphocyte ratio; in addition to age, ca125 and preoperative ultrasound findings.

**Conclusions:**

It seems that neutrophil to lymphocyte and platelet to lymphocyte ratios are useful in predicting borderline ovarian tumors, preoperatively. However, further prospective studies are needed.

## Background

Borderline ovarian tumors (BOTs) are characterized with atypical epithelial proliferation without stromal invasion and they comprise about 10–15 % of primary epithelial ovarian neoplasms [1]. Compared with epithelial ovarian carcinomas (EOCs), BOTs are usually determined at early stages and presented in premenopausal women. Surgery is the mainstay of the treatment and frequently curable with excellent prognosis [2]. Due to the absence of specific preoperative criteria for BOTs, diagnosis is often made by the pathological examinations of the surgical specimens. Findings of imaging methods and ca 125 measurements may indicate suspicious pelvic mass without specifying whether it is invasive or borderline tumor [3]. Distinguishing these tumors (borderline or invasive) from the benign ones is extremely significant in order to skip over the incomplete surgery which results in poor prognosis [4].

Inflammation has a significant role in the development of numerous cancers by various mechanisms such as; up-regulation of cytokines and inflammatory mediators, inhibition of apoptosis, induction of angiogenesis and stimulation of DNA damage. In addition, immune function is compromised by systemic inflammatory response (SIR) mediators which then increase the leukocytes, neutrophils, platelets, C reactive protein (CRP) and fibrinogen concentrations. On the other hand, they decrease the lymphocyte level. Platelets can also be increased by the thrombocytosis induced by cancer. As preoperative inflammatory markers, neutrophil-to-lymphocyte ratio (NLR) and platelet-to-lymphocyte ratio (PLR) have been suggested to be useful for discriminating malignant and benign ovarian tumors. NLR and PLR are noninvasive, easily measured, universally known and cost-effective markers [5–7].

To the best of our knowledge, although NLR and PLR levels (as preoperative markers) have been the subject of many studies concerning ovarian cancers, no specific studies associated with BOTs were found. Therefore, in the present study, we have aimed to investigate the value of using these markers prior to surgery in the patients of BOT.

## Methods

This retrospective study was conducted at the Gynecologic Oncology Department of Çukurova University Medical Hospital in Adana, Turkey and the Gynecologic Oncology Department of Dumlupınar University, Evliya Çelebi Education and Research Hospital in Kütahya, Turkey. The study was approved by The Research Ethics Committee of Çukurova University. Gynecologic Oncology Unit records and pathology reports of the two centers were reviewed retrospectively for the period between 01 January 2002 and 30 December 2015. During this period, 116 cases from Çukurova University (ÇU) and 28 cases from Dumlupınar University (DPU); a total of 144 patients of BOT, and as control group a total of 123 patients of serous cystadenoma (98: from ÇU, 25: from DPU) were detected to be eligible for the study criteria. Clinical data such as age, menopausal status, preoperative ultrasound findings (presence of septa, papillary formation and ascites) ca125 and complete blood counts (CBC) were obtained from the hospital database network and archival files of the patients. Neutrophil, platelet, monocyte, hemoglobin and hematocrit parameters were attained from the CBC profile. These parameters were recorded separately and then NLR and PLR were calculated. The NLR was considered to be the absolute neutrophil count divided by the absolute lymphocyte count, and the PLR was defined as the absolute platelet count divided by the absolute lymphocyte count. Patients whose blood samples were not taken within a week before surgery were excluded from the study population. Also, patients with increased white blood cell count that might reflect a coexisting infection were not included. A story of hematological malignancies or other hematological and autoimmune disorders was the other exclusion criterion. According to the clinical and pathological assessments a total of 267 cases (study group: 144 BOT and control group: 123 serous cystadenoma) comprised the study population. Two groups were compared according to the aforementioned parameters.

Statistical methodology: Continuous variables were presented as mean, median and standard deviation (SD). Chi-square (*χ* ^2^) test evaluated associations between the categorical and continuous variables. Mann-Whitney U and Wilcoxon W tests were performed for the multiple comparisons between variables. The receiver operating characteristic (ROC) curve analysis was carried out to analyze the discriminative role of variables. *P* values were considered statistically significant at *p* < 0.05. SPSS 22.0 Evaluation Version (Statistical Package for Social Sciences, Chicago, IL, USA) software package was used for the statistical analysis.

## Results

The study enrolled a total of 267 patients; 144 with BOT as the study group and 123 with serous cystadenoma (SCA) as the control group. These patients’ distribution was as the following; 116 cases of BOT and 98 cases of SCA from ÇU, 28 cases of BOT and 25 cases of SCA from DPU. Pathological Evaluations of the specimens were performed by expert gynecologic pathologists in the same institutions. Mean age of BOT patients was 41.3 ± 14.7 lower than SCA patients (48.7 ± 16.8) (*p* = 0.000). Most of the BOT patients (72.2 %) and about half of the SCA patients (51.2 %) were premenopausal. Histologic distribution of the BOT cases was as the following; 94 patients (65 %) with serous, 50 (35 %) with mucinous histology. Patients’ clinicopathological characteristics are shown in Table 1.

**Table 1:** Patients’ clinicopathological characteristics

	GROUP		
	Borderline tumor	Serous cystadenoma	
	*N* %	*N* %	*P*
Menopausal status			
Premenopausal	104 (72,2)	63 (51,2)	0,000
Postmenopausal	40 (27,8)	60 (48,8)	
Histological type			
Serous	94 (65)	123 (100,0)	
Mucinous	50 (35)	0 (,0)	
Septa			
No	66 (45,8)	121 (98,4)	0,000
Yes	78 (54,2)	2 (1,6)	
Papilla			
No	33 (22,9)	121 (98,4)	0,000
Yes	111 (77,1)	2 (1,6)	
Ascites			
No	109 (75,7)	123 (100,0)	0,000
Yes	35 (24,3)	0 (,0)	

In the preoperative assessment with ultrasonography; septa pattern was found to be positive in 78 (54.2 %) cases, papilla formation in 111 (77.1 %) and ascites in 35 (24.3 %) of the BOT patients. Nonetheless, all of these parameters were almost negative in the SCA group (*p* = 0,000). While the median of ca125 value was 86 (4–2878) in BOT patients, it was 25 (4–429) in the SCA group (*p* = 0,000).

**Fig. 1:**
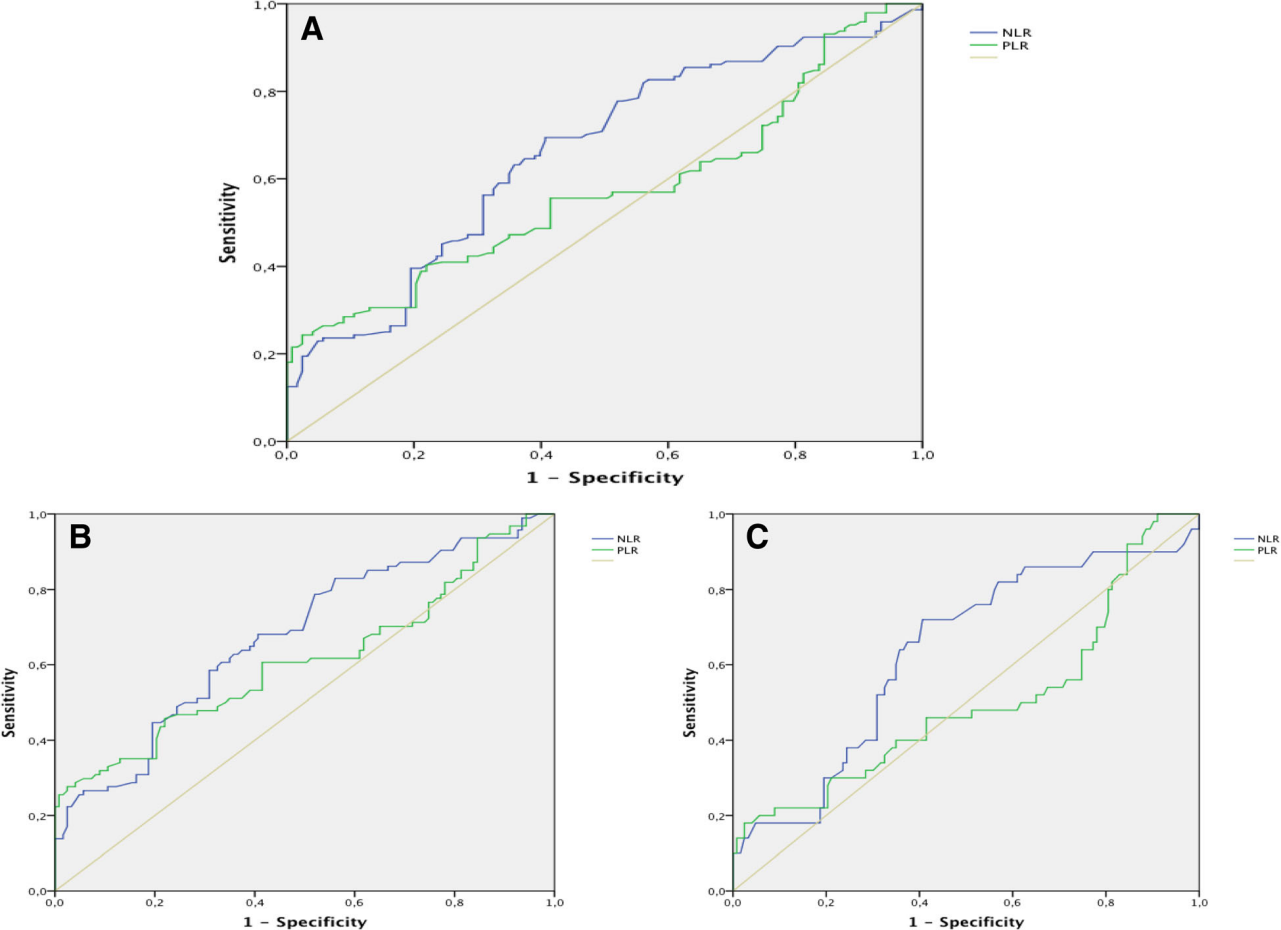
**a** ROC curve for neutrophil to lymphocyte and platelet to lymphocyte ratios between all BOT and SCA cases. **b** ROC curve for neutrophil to lymphocyte and platelet to lymphocyte ratios between SBOT and SCA cases. **c** ROC curve for neutrophil to lymphocyte and platelet to lymphocyte ratios between MBOT and SCA cases

**Fig. 2:**
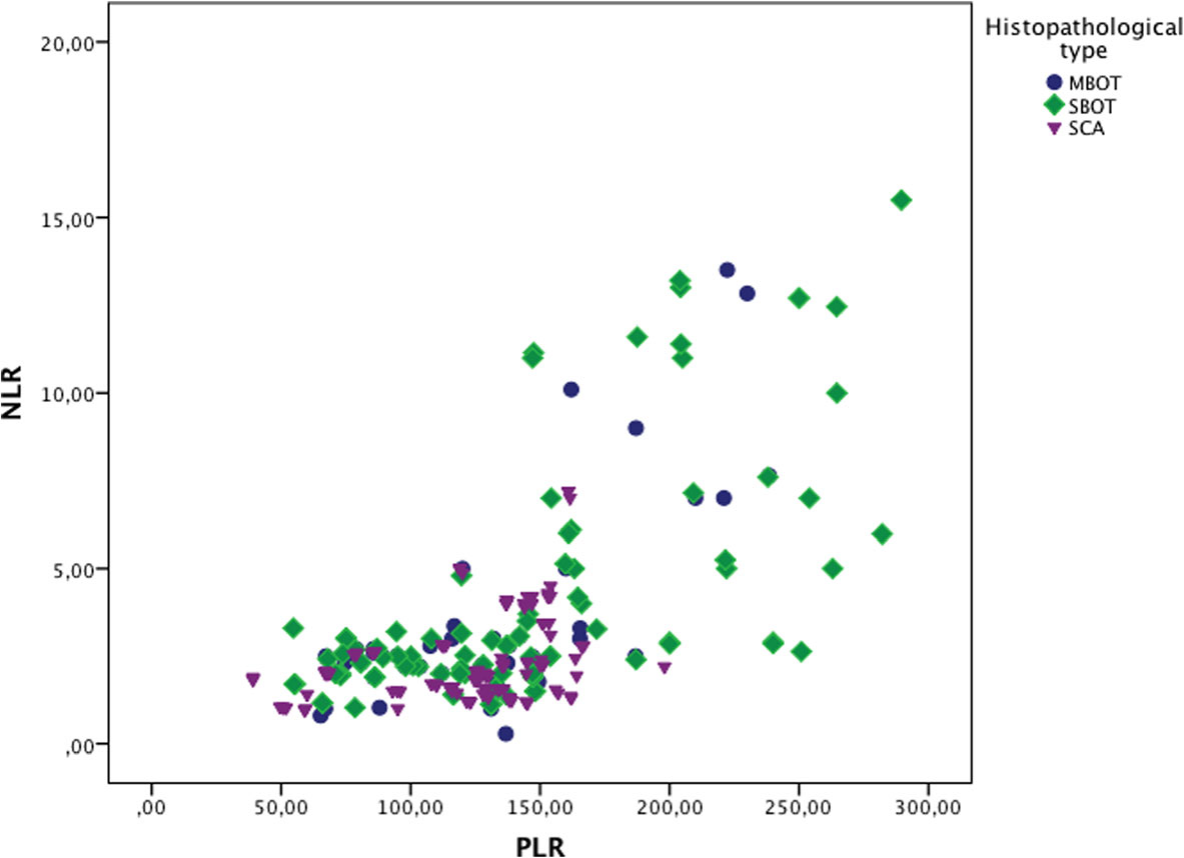
Scatter graph of NLR and PLR according to the histopathological types

From blood count parameters; neutrophil, monocyte, platelet, hemoglobin and hematocrit were evaluated separately. Although, mean and median of the monocyte count of the BOT cases were higher than those of the SCA cases, no statistically significant difference was observed according to the monocyte count between groups (*p* = 0.081). Median of the neutrophils count was 6470 (1040–22300) in the BOT group and 5300 (1700–18700) in the SCA patients. This difference was statistically significant (*p* = 0.001). Also, hemoglobin and hematocrit values were statistically significant when compared between groups (*p* = 0.004). On the other hand, the difference of platelets count between groups appeared to have the tendency to be statistically significant (*p* = 0.05). Median of NLR was 2.5 (0.28–15.5) and 2.0 (0.97–7.2) in the BOT and SCA patients, respectively. This difference regarding the NLR between the study and control groups was detected to be statistically significant (*p* = 0.000). Median of PLR was 133(54.7–289.5) in the BOT cases, whereas it was 128.5 (39–198) in the SCA patients. When compared between groups, results according to the PLR was determined to be statistically significant, also (*p* = 0.039). Comparison between the groups according to the hematologic variables is demonstrated in Table 2. ROC curve analyses were applied to estimate the cut-off values of the NLR and PLR. The recommended cut-off value of NLR in terms of predicting BOT was found to be 2.2 (sensitivity: 66 %, specificity: 60 %). The AUC was 0.661 ± 0.033 (95 % CI: 0.595–0.726) (*p* = 0.000). On the other hand, when a cut-off value of 122.2 was considered for PLR, the sensitivity and specificity were calculated as 57 and 40 %, respectively. The AUC for PLR was 0.573 ± 0.035 (95 % CI: 0.505–0.642, *p* = 0.039) (Fig. 1a). Furthermore, NLR and PLR values of BOT cases were divided according to their histopathological types as serous BOTs (SBOT) and mucinous BOTs (MBOT). While median of NLR and PLR was 2.6 (1.03–15.5) and 136.9 (54.7–289.5) for SBOT, it was 2.48 (0.28–13.5) and 118.42 (65.23–238.4) for MBOT, respectively. Thereafter, these values were compared individually with the NLR and PLR values of the SCA cases (control group), and ROC curve analyses were performed. In the comparison between SBOT and SCA, both of NLR (*p* = 0.000) and PLR (*p* = 0.005) were detected to be with statistically significant discriminative value. The sensitivity and specificity were determined to be 66 % and 60 %, respectively, when a cut-off value by 2.2 of NLR was recommended in identifying SBOT, preoperatively (AUC: 0.675 ± 0.037, 95 % CI: 0.603–0.747). For PLR; a cut-off value of 122.2 was found to be with 62 % of sensitivity and 40 % of specificity in discriminating SBOT cases, preoperatively (AUC: 0.612 ± 0,040, 95 % CI: 0.534–0.690), (Fig. 1b). Whereas; the difference of NLR between MBOT and SCA patients was established to be statistically significant (*p* = 0.006), the difference of PLR between these groups was not (*p* = 0.984). Similar in the all BOT and SBOT patients, the sensitivity and specificity were detected to be 66 % and 60 %, respectively, when a cut-off value by 2.2 of NLR was considered in predicting MBOT, preoperatively (AUC: 0.633 ± 0.047, 95 % CI: 0.541–0.726), (Fig. 1c). A scatter graph of NLR and PLR regarding to the histopathological types is demonstrated in Fig. 2.

**Table 2:** Comparison of patients’ age, ca125 and hematologic variables between the groups

Parameter	GROUP								*P*
	Borderline tumor				Serous cystadenoma				
	Mean	Standard Deviation	Median	Minimum-Maximum	Mean	Standard Deviation	Median	Minimum-Maximum	
Age	41,3	14,7	39	14–80	48,7	16,8	49	17–84	0,000
ca125	170,5	301,5	86	4–2878	32,9	50,0	25	4–429	0,000
NLR	3,8	3,3	2,50	0,28–15,5	2,3	1,3	2,00	0,97–7,20	0,000
PLR	138,4	55,7	133	54,7–289,5	119,7	34,1	128,50	39–198	0,039
Monocyte	6101,6	3375,2	6345	1600–40000	5498,7	1866,6	4890	560–9030	0,081
Neutrophil	7694,6	4528,6	6470	1040–22300	5892,7	3013,7	5300	1700–18700	0,001
Platelet	293186,2	90934,4	294000	90000–531000	277000	74069,7	275000	100000–584000	0,050
Hemoglobin	12,5	8,6	11,90	7,4–11,25	11,2	1,8	11,20	7,3–15	0,004
Hematocrit	38,3	28,8	35,75	25,9–46	38,9	36,9	33,70	24,4–47,6	0,004

## Discussion

Preoperative assessment of an ovarian mass includes gynecologic examinations, imaging methods and tumor markers. Despite of all these tools, postoperative pathological evaluation is mandatory for the absolute diagnosis. Therefore, efforts are still made in order to obtain the ideal prediction of the mass nature. Risk of malignancy index (RMI) which includes ca125, ultrasound findings and menopausal status have been developed for this purpose. The increasing evidences emphasize the importance of inflammation in the initiation, promotion, invasion and metastasis of cancer [5]. It was found that neutrophils increased and lymphocytes relatively decreased as a result of the systemic inflammatory response [8]. Numerous studies have demonstrated the association between NLR and PLR with the ovarian cancers [4–6, 8]. In the current study, the association between NLR and PLR with BOTs has been investigated. A statistically significant impact for both of preoperative NLR (*p* = 0,000) and PLR (*p* = 0,039) in distinguishing BOTs from simple ovarian serous cysts was suggested, in this study. Beside, hemoglobin and hematocrit values, neutrophils, monocytes and platelets counts were evaluated and then compared separately between the groups. From these variables, merely monocytes count was not in a statistically significant manner. On the other hand, classic markers such as age, ca125 and ultrasound findings (septa, papillary pattern and ascites) were found to have statistically significant difference between the groups.

Topçu et. al. reported that they found statistically significant difference in term of age, diameter of the mass, ca125 levels, preoperative PLR and platelet count between the benign and malignant ovarian masses, whereas they didn’t find statistically significant effect in term of NLR [4]. In the study by Yıldırım et. al. 316 benign and 253 malignant cases were assessed and preoperative NLR, PLR, and monocyte values were established to be higher in the malignant cases. Authors stated that NLR and PLR combined with CA-125 can be useful for benign-malignant differentiation of the ovarian masses [8]. Cho et al. indicated that NLR is significantly increased in ovarian cancer cases. Moreover, it was supposed that NLR can identify CA-125-negative cases and predict poor outcome [9]. NLR was showed to be associated with aggressive disease and poor survival in a retrospective study by Williams et al. of 519 ovarian carcinoma cases [10]. PLR were also found to be elevated and correlated with poor prognosis and low survival rates in ovarian cancers by the study of Asher et. al. [11]. Furthermore, Zhang and colleagues presumed that preoperative PLR was superior to the other SIR markers (CA-125, NLR, fibrinogen, CRP, and albumin) as a predictor of survival in ovarian cancer patients [7]. Although several studies investigated the association between preoperative NLR and PLR with invasive ovarian cancers, to the best of our knowledge, there were no reported studies in the literature which researched these markers in the BOTs. Hence, it is deemed necessary to conduct the present study. A simple blood count is sufficient to provide these markers. These easily measured hematological variables were considered to be useful in discriminating BOTs from benign cysts preoperatively, in this study. Especially, NLR and PLR were fixed to be with an acceptable sensitivity and specificity values in terms of predicting borderline ovarian tumors, preoperatively. In addition, the difference between the study and control groups according to the classic predictors (age, ca125, ultrasound findings such as septa, papilla and ascites) was found to be statistically significant. Therefore, a novel model consisting of these classic predictors in combined with NLR and PLR values may be promising in identifying ovarian masses.

## Conclusion

In conclusion, it seems that neutrophil to lymphocyte and platelet to lymphocyte ratios are useful in predicting borderline ovarian tumors, preoperatively. However, further prospective, large simple-size studies should be conducted on this subject.
